# Emotion Differentiation and Youth Mental Health: Current Understanding and Open Questions

**DOI:** 10.3389/fpsyg.2021.700298

**Published:** 2021-08-06

**Authors:** Erik C. Nook

**Affiliations:** ^1^Department of Psychology, Harvard University, Cambridge, MA, United States; ^2^Department of Psychiatry, NewYork-Presbyterian Hospital/Weill Cornell Medical Center, New York, NY, United States

**Keywords:** emotion differentiation, development, mental health, psychopathology, adolescence

## Abstract

A growing body of research identifies emotion differentiation—the ability to specifically identify one’s emotions—as a key skill for well-being. High emotion differentiation is associated with healthier and more effective regulation of one’s emotions, and low emotion differentiation has been documented in several forms of psychopathology. However, the lion’s share of this research has focused on adult samples, even though approximately 50% of mental disorders onset before age 18. This review curates what we know about the development of emotion differentiation and its implications for youth mental health. I first review published studies investigating how emotion differentiation develops across childhood and adolescence, as well as studies testing relations between emotion differentiation and mental health in youth samples. Emerging evidence suggests that emotion differentiation actually *falls* across childhood and adolescence, a counterintuitive pattern that merits further investigation. Additionally, several studies find relations between emotion differentiation and youth mental health, but some instability in results emerged. I then identify open questions that limit our current understanding of emotion differentiation, including (i) lack of clarity as to the valid measurement of emotion differentiation, (ii) potential third variables that could explain relations between emotion differentiation and mental-health (e.g., mean negative affect, IQ, personality, and circularity with outcomes), and (iii) lack of clear mechanistic models regarding the development of emotion differentiation and how it facilitates well-being. I conclude with a discussion of future directions that can address open questions and work toward interventions that treat (or even prevent) psychopathology.

## Introduction

Some people easily label their emotional experiences using precise terms (e.g., differentiating when they feel “frustrated” from when they feel “disappointed”), but others struggle to make such fine-grained distinctions and instead focus merely on whether they feel “good” or “bad” in any given moment. This individual difference is referred to as *emotion differentiation* or *emotional granularity*. Several studies have demonstrated that people with higher emotion differentiation tend to have better mental health (see Kashdan et al., 2015; Smidt and Suvak, 2015; Trull et al., 2015; Hoemann et al., 2020a; Thompson et al., 2021 for reviews and O’Toole et al., 2020; Seah and Coifman, 2021 for meta-analyses). A substantial body of research in adult samples now shows that emotion differentiation scores are associated with healthier and more effective responses to intense negative emotions (Barrett et al., 2001; Tugade et al., 2004; Kashdan et al., 2010; Pond et al., 2012; Zaki et al., 2013; Kalokerinos et al., 2019; Ottenstein, 2020) and that emotion differentiation scores tend to be lower in adults experiencing several forms of psychopathology (e.g., depression, anxiety disorders, eating disorders, schizophrenia, autism, and borderline personality disorder; Decker et al., 2008; Demiralp et al., 2012; Erbas et al., 2013; Dixon-Gordon et al., 2014; Kashdan and Farmer, 2014; Kimhy et al., 2014; Tomko et al., 2015; Mikhail et al., 2020). Together, this body of research suggests that the ability to specifically identify one’s emotions bolsters adaptive emotional responding and protects against psychopathology.

To date, however, very few studies have examined emotion differentiation in developmental samples, constraining our knowledge of this phenomenon almost entirely to adult populations. This is a major gap in understanding, especially considering that childhood and adolescence are active windows of change in several social and emotional processes (Guyer et al., 2016; Somerville and McLaughlin, 2018; Nook and Somerville, 2019). Across childhood, people gradually learn how to define emotion words, to accurately label emotional facial expressions, to predict specific emotional responses from contextual settings, and to manage their emotional responses (Baron-Cohen et al., 2010; Widen, 2013; Silvers et al., 2017; Lagattuta and Kramer, 2019; Nook et al., 2020). In fact, the abilities to conceptualize one’s own and others’ emotions show protracted development, continuing to mature into adolescence (Dumontheil et al., 2010; Sebastian et al., 2012; Nook et al., 2017, 2020), and adolescence is a period of the lifespan where neural, hormonal, and social changes bring about increased stress and negative emotion compared to childhood (Larson and Ham, 1993; Larson et al., 2002; Romeo and McEwen, 2006; Steinberg, 2015). These social and emotional transitions make adolescence a period of increased risk for the onset of psychopathology, with an estimated 50% of all mental illnesses arising before age 18 (Kessler et al., 2005; Sawyer et al., 2012).

Given that transitions from childhood to adolescence are times of both substantial emotional change *and* increased risk for psychopathology, it is imperative that we clearly understand how youth might best manage their emotional experiences and healthfully navigate this period of their lives. This renders greater understanding of the development of emotion differentiation extremely important, as it could provide insight into how normative changes in affective processes relate to increased risk for psychopathology in adolescence. Such insight could then guide psychological interventions that protect youth from psychopathology. Fortunately, researchers have begun to examine how emotion differentiation develops across age, as well as how emotion differentiation scores relate to well-being in youth samples. The first goal of this paper is to synthesize this burgeoning literature, taking stock of elements of commonality and areas for future growth in our understanding of the development of emotion differentiation and how it relates to well-being in youth.

However, the scientific study of emotion differentiation (in both adult and developmental samples) is limited by several open questions, including: (i) concerns about the construct validity of current emotion differentiation measures, (ii) the presence of third variables that might explain existing relations between emotion differentiation and mental health (e.g., IQ, mean negative affect, circularity with outcome measures), and (iii) a lack of clear mechanistic models for how emotion differentiation develops and how it facilitates mental health. These topics have received some attention in the adult literature (Trull et al., 2015; Dejonckheere et al., 2019; Hoemann et al., 2020a; Thompson et al., 2021), but not in developmental populations. Therefore, the second goal of this paper is to clearly describe these limitations and discuss how they manifest in studies of emotion differentiation in youth. Even though research on youth emotion differentiation is in its infancy, there are several reasons to conduct a review at this point. First, identifying (in)consistencies in methods and results across emerging studies can provide insight into the stability of effects and generate hypotheses of potential moderating factors. Second, as noted above, studies on the development of emotion differentiation have lagged behind studies on adults, suggesting that clearly articulating the impact of a developmental approach to this phenomenon could stimulate future research in this area. Third, identifying and summarizing key open questions can improve future studies by laying out an agenda of research questions in need of study that can together work toward a clear scientific account of emotion differentiation (see Box 1 for a summary).

**Box 1. Key lessons, open questions, and future directions for research on youth emotion differentiation.**

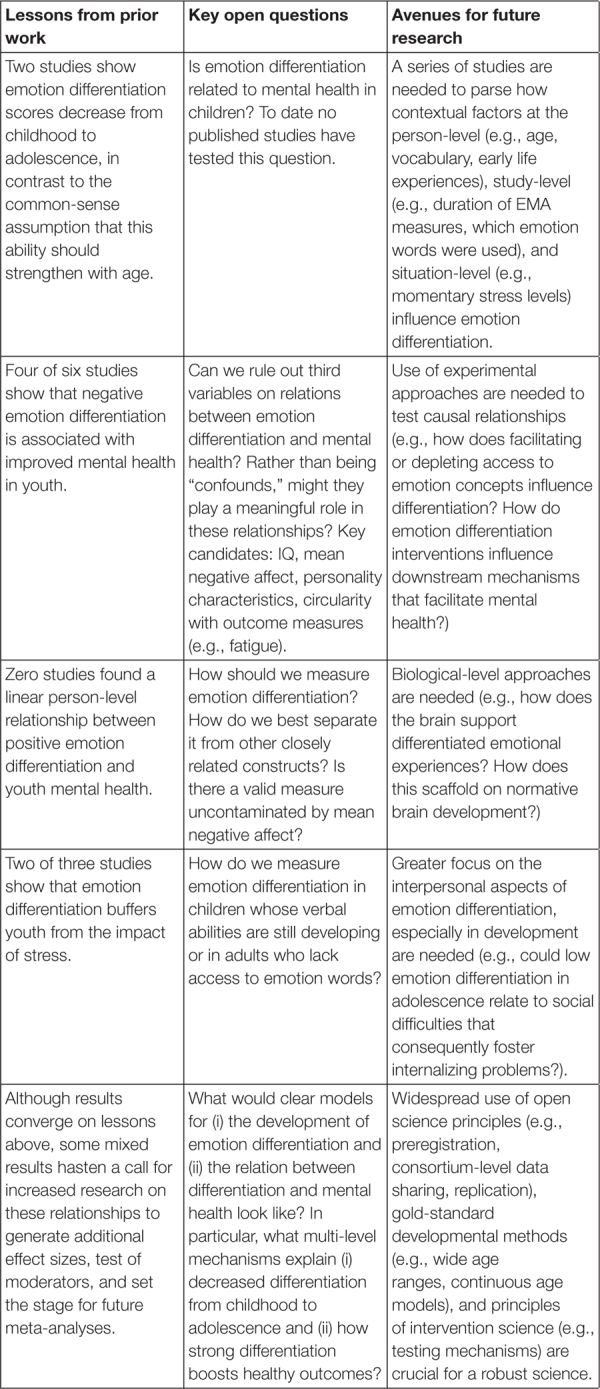

This paper is organized into five sections, which together aim to articulate what we do and do not know about the development of emotion differentiation and youth mental health. The first section “Carving the Subject Matter” defines emotion differentiation and carves it away from other related phenomena. The second section “Emotion Differentiation Across Age” then synthesizes published studies examining how emotion differentiation scores vary across age to chart what we have learned about its shift from childhood into adulthood. The third section “Emotion Differentiation and Youth Mental Health” reviews studies showing how emotion differentiation scores relate to mental health variables in youth samples (i.e., participants younger than age 18). The fourth section “Key Questions for the Study of Emotion Differentiation in Youth” then identifies key open questions that limit our understanding of how emotion differentiation relates to mental health in developmental samples. The final section “Discussion and Future Directions” provides a general discussion of the paper’s topics and outlines ideas for future research.

## Carving the Subject Matter

Given the diversity of emotion constructs that exist in the literature, it is important to define what I mean by emotion differentiation and outline the scope of the current review. The field has generated a wide array of constructs that seek to quantify how aspects of affective experience differ across individuals (e.g., emotion awareness, emotional intelligence, emotional clarity, emotional intensity, emotional complexity, emotional instability, emotion comprehension, emotional inertia, emotion abstraction, alexithymia, and emodiversity (Sifneos, 1973; Lane and Schwartz, 1987; Kang and Shaver, 2004; Salovey and Grewal, 2005; Waugh et al., 2011; Boden et al., 2013; Quoidbach et al., 2014; Koval et al., 2016; Nook et al., 2020). Scholars debate the best way to organize these different constructs (Grühn et al., 2013; Thompson et al., 2021), with some arguing that they can be meaningfully integrated into a framework of “emotional expertise” (Hoemann et al., 2020a), while others argue that they are largely redundant in the prediction of mental health in adults (Dejonckheere et al., 2019). Regardless, these constructs are associated with psychological health in both youth and adult samples (e.g., Zeidner et al., 2012; Boden and Thompson, 2015; Trull et al., 2015; Bailen et al., 2019), and we have data on how some of them vary across age (Bailen et al., 2019; Haas et al., 2019; Reitsema et al., 2021). Although the questions of how these constructs relate to each other and how they all develop are interesting and fruitful directions of research, the current paper engages only with emotion differentiation, which is defined as *how specifically people identify their emotional experiences* (see Barrett et al., 2001; Kashdan et al., 2015).

This paper focuses on studies that operationalize emotion differentiation in its classic formulation as the intercorrelation between self-reported emotional experiences, which I refer to as *emotion differentiation scores*. The earliest studies of emotion differentiation asked participants to rate their emotions at the end of each day on 5-point scales (Barrett et al., 2001) and calculated the average of all pairwise correlations between emotion ratings. The logic of this approach is that participants who struggle to conceptualize emotions in specific emotional terms (instead representing their affect as merely “good” or “bad”) will consistently endorse similar emotional states across days. For example, at the end of a “bad” day, they will provide similarly intense ratings of fear, anger, and sadness, and on a “less bad” day, they will report slightly less intense ratings of all of these emotions. By contrast, people with high emotion differentiation will select unique profiles of emotion terms that specifically describe their emotional reactions on each day (e.g., providing high fear and anger ratings on a day that elicited those specific emotions but high anger and sadness ratings on a different day). Correlations between emotion ratings will be high for the first kind of individual and low for the second kind of individual, meaning that low correlation coefficients between emotions are taken as an indication of high emotion differentiation.

This method is still widely used to measure emotion differentiation, with a few key advances. Researchers have increased the number of emotion ratings participants complete each day (i.e., using *ecological momentary assessment* [EMA] methods), and laboratory measures have also been introduced in which emotion ratings are made in response to standardized image sets (Erbas et al., 2014; Nook et al., 2018). Additionally, intraclass correlations (ICCs) are often used instead of pairwise correlations (Kalokerinos et al., 2019). Nonetheless, the logic of these methods remains identical: High consistency in emotion ratings across instances indicates poor differentiation of one’s affect. Studies using this method will be reviewed in the current paper.

When possible, researchers apply these methods separately for ratings of “positive” and “negative” emotions, leading to measures for positive emotion differentiation (i.e., how specifically people differentiate emotions like happiness, gratitude, excitement, and amusement) and negative emotion differentiation (i.e., how specifically people differentiate emotions like sadness, anger, fear, and disgust). Positive and negative emotion differentiation are computed separately so that each score can represent how specifically people identify emotions *within* a class of emotions that share a similar valence, though some research has investigated overall differentiation using an ICC across both positive and negative emotions (e.g., Kimhy et al., 2014). Researchers have also recently developed methods to quantify how specifically people differentiate emotions at the *moment-level*, not just at the *person-level* (Tomko et al., 2015; Erbas et al., 2018, 2021). These studies offer compelling evidence that emotion differentiation varies *within* individuals and that these oscillations share interesting relationships with outcomes. Unfortunately, because these methods have not yet been investigated in youth, no studies using this approach are reviewed. Similarly, although researchers have attempted to measure emotion differentiation through self-report questionnaires (e.g., the Range and Differentiation of Emotional Experience Scale; Kang and Shaver, 2004), convergent validity with the canonical ICC approach has not been established. As such, the current paper will focus only on studies that use repeated assessments of experienced emotions.

## Emotion Differentiation Across Age

If being able to specifically identify emotions helps people manage them, then it seems important to understand the normative trajectories through which this affective skill develops. Indeed, the framework of developmental psychopathology (Sroufe and Rutter, 1984; Cicchetti and Sroufe, 2000; Cicchetti and Rogosch, 2002) emphasizes four key steps in understanding and preventing psychological disorders: (i) identify how psychological processes typically emerge across age, (ii) document differences between these normative trajectories and trajectories indicative of psychopathology, (iii) elucidate the mechanisms that produce these diverging trajectories, and (iv) develop interventions that bring pathological trajectories into alignment with healthy development. To date, two published studies have investigated the typical developmental trajectory of emotion differentiation (one assessing negative emotion differentiation and one assessing both positive and negative emotion differentiation, Table 1).

**TABLE 1:** Published papers investigating emotion differentiation in youth.

Authors	Year	Usable *N*	Age range	Emotion differentiation measure				Relationships with:				Mean affect control?
				Method	Valence	Stimuli Duration	Emotions	Age	Psychopathology	Stress buffering	Other	
Nook, Sasse, Lambert, McLaughlin, and Somerville	2018	143	5–25	Lab task	Negative	20 negative images	Angry, disgusted, sad, scared, and upset	U-shaped relationship	–	–	–	Yes (from lab task)
Starr, Shaw, Li, Santee, and Hershenberg	2020	233	14–17	EMA	Negative	4 prompts/day for 7 days	Anxious, sad, annoyed, angry, and worn-out	Linear negative relationship	Significant negative relationship with depression symptoms in community sample	–	Significant negative relationships with parental depression, parental attachment, and parenting style	Yes (from EMA)
					Positive		Happy, proud, cheerful, lively, and joyful	Not significant (negative direction)	Not significantly related to depression symptoms in community sample	–	Significant negative relationships with parental depression and parental attachment	
Erbas, Ceulemans, Boonen, Noens, and Kuppens	2013	18 ASD + 26 TD	15–19	Lab task	Negative	20 negative images	Fear, worry, anxiety, nervousness, anger, irritation, disgust, rage, shame, guilt, regret, embarrassment, sadness, loneliness, unhappiness, depression, jealous, envious, and two Dutch words for inferior	–	Significantly lower in participants diagnosed with ASD compared to controls (one-tailed)	–	–	No
Lennarz, Lichtwarck-Aschoff, Timmerman, and Granic	2018	86	Not given (*M* = 14)	EMA	Negative	22 prompts/weekend for 2 weekends (4 on Friday, 9 on Saturday, 9 on Sunday)	Jealous, anxious, ashamed, irritated, worried, angry, guilty, sad, and lonely	–	Not significantly related to depression symptoms in community sample	–	Significant negative association with mean negative affect and implicit theories of emotion	No
					Positive		Happy, cheerful, satisfied, relaxed, and proud.	–	Not significantly related to depression symptoms in community sample	–	Significant positive association with mean positive affect	
Starr, Hershenberg, Shaw, Li, and Santee	2020	233	14–17	EMA	Negative	4 prompts/day for 7 days	Anxious, on edge, uneasy, sad, hopeless, discouraged, angry, resentful, annoyed, fatigued, worn out, and exhausted		Significant relationship with depression symptoms concurrently and 1.5 years later in community sample*	Significantly buffered relationship between stressful events and concurrent depression symptoms, depression symptoms 1.5 years later, and momentary depressed affect	–	Yes (from EMA)
					Positive		Happy, proud, cheerful, lively, and joyful	–	Not significantly associated with depression symptoms in community sample	Significantly buffered relationship between daily hassles and concurrent depressed affect, but no longer significant when controlling for negative emotion differentiation	–	
Schreuder et al.	2020	401	15–18	EMA	Negative	10 prompts/day for 6 days	Lonely, anxious, irritated, listless, suspicious, down, insecure, guilty	–	Significantly associated with good prognosis (global severity score below cutoffs) 1 year later in community sample of twins**	No interaction between negative emotion differentiation and stressful events in predicting prognosis 1 year later	–	Yes (from EMA)
Nook, Flournoy, Rodman, Mair, and McLaughlin	2021	30	15–17	Lab task	Negative	20 negative images	Angry, ashamed, disgusted, sad, and scared	–	Not significantly associated with depression or anxiety in community sample	Significantly buffers relationship between perceived stress and depressed affect (moment level), as well as relationship between stressful events and anxiety symptoms (month level)	–	Yes (from lab task)
					Positive	20 positive images	Calm, excited, happy, inspired, and interested	–	Not significantly associated with depression or anxiety in community sample	Significantly buffers relationship between perceived stress and depressed affect (moment level)	–	

*EMA, ecological momentary assessment; ASD, autism spectrum disorder; TD, typically developing.*

All studies used the ICC method for computing emotion differentiation scores.

*Prospective association no longer significant when controlling for baseline depression symptoms and mean negative affect.

**No longer significant after controlling for mean negative affect.

One study asked a cross-sectional sample of 143 participants aged 5–25 to complete a laboratory measure of negative emotion differentiation (Nook et al., 2018). Participants viewed 20 negative images and rated how angry, sad, disgusted, scared, and upset they felt in response to each image, and an emotion vocabulary test was used to exclude participants who didn’t comprehend these terms (Nook et al., 2020). Emotion differentiation scores (i.e., the inverse of intraclass correlations on these ratings) revealed an inverted-U relationship with age: Negative emotion differentiation scores fell from childhood to around age 15 before rising again into young adulthood. Somewhat surprisingly, this suggested that young children were actually *better* at differentiating negative emotions than adolescents. Further analyses revealed that children were more likely to report experiencing only one emotion at a time compared to older participants and that this tendency statistically explained why emotion differentiation scores fell from childhood to age 15. This finding converged with previous research showing that children tend to report emotions one-at-a-time (Harter and Buddin, 1987; Wintre and Vallance, 1994; Larsen et al., 2007). As such, the current study provided initial evidence for a non-linear developmental trajectory for emotion differentiation.

These results were partially replicated in a study of 233 adolescents aged 14–17 (Starr et al., 2020b). This study used an EMA method in which participants received four prompts each day for seven days with a survey asking them to report how strongly they felt five negative emotions (i.e., anxious, sad, annoyed, angry, worn-out) and five positive emotions (i.e., happy, proud, cheerful, lively, joyful) on 7-point scales. ICCs were again used to quantify negative and positive emotion differentiation. Here the researchers only found a significant linear decrease in negative emotion differentiation scores across age, and tests for non-linear trajectories were not significant. Positive emotion differentiation scores also showed an overall negative relationship with age, but this did not reach significance. Even though the tendency to report feeling only one negative emotion at a time was related to both negative and positive emotion differentiation scores, it did not vary across age.

Although these two studies used different methods (laboratory vs. EMA measurement of emotion differentiation and broad vs. narrow age range), they converge on the intriguing finding that younger individuals actually construct negative emotions in a *more* differentiated fashion than older individuals. This may seem surprising, given notions that children tend to focus on whether emotion concepts are merely “positive or negative” and that additional complexity in emotion concepts emerge with age (Pons et al., 2004; Widen and Russell, 2008; Widen, 2013; Nook et al., 2017; Morningstar et al., 2019). However, the story seems more complex than the takeaway that children are expert emotion differentiators. Instead, children may construct emotional experiences in entirely different ways than adults. Children tend to report experiencing emotions one at a time, potentially suggesting that they believe emotions are mutually exclusive mental states. This reveals an important nuance about the measurement of emotion differentiation, as children and adults might both achieve the same differentiation score but have entirely different “routes” to obtaining this score: Whereas children clearly identify what they are feeling by endorsing a single emotion at a time, adults can differentiate emotions even while multiple are co-experienced simultaneously. Adolescents appear to be somewhere in the middle of these two developmental processes, struggling to differentiate newly co-experienced emotions. Interestingly, other lines of research also show that youth report *greater* difficulty in labeling and describing their emotions as they age from childhood to adolescence (Haas et al., 2019; Weissman et al., 2020). These findings may all reflect overlapping psychological processes that render emotions more difficult to identify in adolescence, but future research is needed to gain clarity on relations between these constructs, as well as the down-stream impacts of this developmental shift on emotion regulation and mental health.

The two studies reviewed above differed in what they suggest about the trajectory of negative emotion differentiation from age 15 onward. The first suggested an increase across age, whereas the second did not find this U-shaped pattern. This could be explained by the age restriction of the second study, which may not have included enough data from older participants to capture the increase across this window. That said, the four published studies on emotion differentiation in older adults provide mixed results: Older adults’ emotion differentiation scores are sometimes higher than (Mankus et al., 2016), sometimes lower than (Brose et al., 2015) and sometimes equivalent to (Grühn et al., 2013; Mikkelsen et al., 2020) younger adults. This heterogeneity in effects either suggests that emotion differentiation is largely unchanging over this age range (i.e., positive or negative correlations emerge in a given study merely due to sampling error) or that there are important study-level factors that systematically influence these results.

## Emotion Differentiation and Youth Mental Health

Even though studies show that emotion differentiation scores track mental health in adult samples, this does not necessarily imply that the same relationship exists in younger samples, especially given that emotion differentiation scores vary normatively across age. A handful of studies have investigated both simple associations between emotion differentiation scores and mental health in youth samples as well as more complex questions of whether emotion differentiation offers resilience to youth when they face stressful life experiences (Table 1).

### Associations Between Emotion Differentiation and Mental Health

Erbas et al. (2013) tested whether emotion differentiation differed between 18 adolescents with autism spectrum disorders (ASD, ages 15–19) and 26 typically developing adolescents (ages 15–18). Both groups completed a laboratory-based assessment of negative emotion differentiation in which they rated how strongly they felt 20 negative emotions in response to 20 negative images. Emotion differentiation scores computed from intraclass correlations were significantly lower in participants with ASD compared to controls. A second measure in which participants sorted 20 emotion words into piles also showed that participants made fewer piles than controls—suggesting less differentiation—though the result was on the margin of significance (*p* = 0.06). Hence, this study provided initial evidence that negative emotion differentiation is lower in adolescents with ASD.

A number of studies have examined how youth emotion differentiation relates to symptoms of depression. Lennarz et al. (2018) assessed both positive and negative emotion differentiation in 86 adolescents (72 included in a subset of analyses, *M*_*age*_ = 14). Participants completed EMA measures over two weekends (44 prompts total) and ICCs were applied to compute emotion differentiation scores. Negative emotion differentiation scores were negatively correlated with (i) the average intensity of negative affect over the EMA periods, (ii) self-reported propensity to experience negative emotions in the weeks between sampling periods, and (iii) having a “fixed mindset” regarding negative emotions (i.e., higher emotion differentiation scores were associated with more adaptive implicit theories of emotions, Tamir et al., 2007). By contrast, positive emotion differentiation scores were only associated with the average intensity of positive emotions during EMA periods, but this relationship was rendered non-significant when controlling for gender. Interestingly, neither positive nor negative emotion differentiation scores were related to self-reported depressive symptoms, though this may be due to the fact that the sample rarely endorsed symptoms (*M* = 0.35).

The Starr et al. (2020a) paper described above also included measures of both participant and parent mental health. Adolescents with higher negative emotion differentiation scores endorsed significantly fewer depressive symptoms, and a negative (but non-significant) relationship also emerged for positive emotion differentiation scores. Interestingly, adolescents with higher positive and negative emotion differentiation scores also had *parents* who were less depressed, and they reported being more securely attached to their parents. Finally, parents’ self-report of parental style was significantly related to negative emotion differentiation scores such that parents who were less authoritarian had adolescents with higher negative emotion differentiation scores. Although one cannot infer causality or directionality from these correlational results, they nonetheless highlight the fact that youth emotion differentiation emerges in the context of family environments such that parental well-being and parenting styles may shape or be shaped by adolescent emotion differentiation.

Three additional studies investigated how emotion differentiation relates to internalizing symptoms in youth (Schreuder et al., 2020; Starr et al., 2020a; Nook et al., 2021a), and they also included analyses testing whether emotion differentiation buffers the impact of stress on youth (reviewed below). In Starr et al. (2020a), 233 participants aged 15–17 completed interviews assessing depression symptoms (Kaufman et al., 1997) both immediately before EMA measures used to assess emotion differentiation (labeled T1) as well as 1.5 years later (labeled T2). Negative emotion differentiation scores were significantly related to both T1 depression symptoms and T2 depression symptoms 1.5 years later. However, the prospective relationship with T2 symptoms was no longer significant when controlling for T1 depression scores and mean negative affect endorsed in EMA sampling. Positive emotion differentiation scores were not significantly related to depression symptoms at either timepoint.

Nook et al. (2021a) conducted an intensive longitudinal study of 30 adolescent girls aged 14–17 and examined relations between emotion differentiation assessed using a laboratory-based picture rating task and self-reported measures of depression and anxiety symptoms. Although positive and negative differentiation scores were negatively correlated with internalizing symptoms, they did not reach significance at the between-persons level. This was likely due to the small *N*, as the study was optimized to detect within-persons, rather than between-persons effects (see next section for significant within-person buffering effects).

Finally, Schreuder et al. (2020) examined prospective relations between emotion differentiation and broad indices of psychopathology over a 1-year period in 401 participants aged 15–18. They used a slightly different measure of psychopathology in which they administered the SCL-90 (Arrindell and Ettema, 1986) at the beginning and end of the study period and categorized participants into “good” or “bad” prognosis depending on how these scores had changed over time (Schauenburg and Strack, 1999). They found that negative emotion differentiation scores assessed by 6 days of EMA were significantly related to prognosis 1 year later, but not after controlling for mean negative affect.

Together, these studies extend research on the positive correlates of emotion differentiation in adults to developmental samples, with 4 of 6 finding a relationship between emotion differentiation and psychopathology (and the other 2 either finding that emotion differentiation tracks a broader index of negative affect or that it buffers the impact of stress). This suggests that emotion differentiation is indeed associated with youth mental health. However, few caveats must be kept in mind regarding this association. First, all relations with psychopathology only exist for negative emotion differentiation, and no significant relations emerge between psychopathology and positive emotion differentiation. Unfortunately, this means that the number of significant relations falls to the much less rosy 4 out of 10 when the total number of comparisons is taken into account. Although there are indeed legitimate reasons why some of these relations were null (e.g., low sample symptom prevalence, low between-subject power due to an intensive within-person design, positive emotion differentiation scores are known to be less consistently associated with outcomes; Liu et al., 2020), a 40% significance rate raises concerns about the stability of the effect. As such, further evidence is needed to gain confidence in the association between mental health and negative emotion differentiation. Additionally, although it is compelling to find in two studies that emotion differentiation has *predictive* relations with psychopathology, this relationship became non-significant after controlling for baseline symptoms and/or mean negative affect. This raises questions about the unique contribution of differentiation on youth mental health, as has been raised in the adult literature (Dejonckheere et al., 2019).

Taken together, this review suggests that negative emotion differentiation *may* track psychopathology in youth, but the evidence base is not as strong as for the adult literature. In fact, the evidence summarized here might indicate that differentiation is helpful in adolescence but with a smaller effect than in adulthood. A meta-analysis following additional data collection would greatly aid in estimating the stability and size of these relationships. Meta-analytic approaches would also allow us to clarify whether moderators might explain when we would or would not expect significant relations. As such, additional studies that estimate relations between differentiation and youth mental health are sorely needed. Most notably, there are *no* studies testing how emotion differentiation relates to *child* mental health, as no studies focused on participants less than age ∼14. Even if it seems reasonable to expect that emotion differentiation is helpful in managing emotions in childhood—as it is in adults—this remains an important open empirical question for the field to address. This is especially true considering the evidence reviewed previously showing that there are many facets of emotion construction that differ between childhood, adolescence, and adulthood. Similarly, only one study selected participants based on diagnosed levels of psychopathology (Erbas et al., 2013) and all others used community samples. Although community samples often include symptomatic individuals (e.g., at T2 in Starr et al., 2020a, 37% of participants reported clinically significant symptoms and 16% met criteria for major depressive disorder), future research should verify that these relationships exist when directly comparing youth with and without elevated symptom levels (as has been done in adults; e.g., Demiralp et al., 2012).

### Emotion Differentiation as a Buffer Against Stress

A long history of scholarship has associated stressful life experiences (i.e., situations in which people experience significantly unexpected and/or threatening events that tax their available coping resources; Lazarus and Folkman, 1984) with heightened risk of psychopathology (Larson and Ham, 1993; Grant et al., 2006; McLaughlin and Hatzenbuehler, 2009; Michl et al., 2013). However, if emotion differentiation does indeed facilitate emotion regulation, then differentiation may be especially helpful in buttressing individuals during stressful experiences, when emotions run high. Indeed, relatively early studies of emotion differentiation in adults demonstrated that emotion differentiation scores were associated with healthier coping strategies specifically when negative emotions were elevated (Kashdan et al., 2010; Zaki et al., 2013), and these results have been replicated and extended more recently (Starr et al., 2017; Liu et al., 2020). To date, three published studies introduced above have extended this line of research into developmental populations.

Starr et al. (2020a) tested whether emotion differentiation buffered the impact of stress on psychopathology at two levels. First, within the EMA method that assessed emotion differentiation, participants also indicated if any stressful events (defined as daily hassles across several life domains; Cohen et al., 2005) had occurred since the last prompt and rated the intensity of this event. Both positive and negative emotion differentiation scores moderated the relationship between these stressors and concurrent reports of depressed mood (i.e., the average EMA rating of feeling sad, hopeless, and discouraged), though the interaction for positive emotion differentiation scores was no longer significant when controlling for negative emotion differentiation scores. Nonetheless, these results suggest that adolescents with higher emotion differentiation felt less depressed in response to daily stressors compared to adolescents with low emotion differentiation, just as has been found in two studies with adult participants (Starr et al., 2017). Second, this moderation was also discovered outside of EMA measures. Participants completed interviews assessing stressful life events (Hammen et al., 2000) and depression symptoms (Kaufman et al., 1997) immediately before the EMA assessment (T1) and approximately 1.5 years later (T2). Somewhat astoundingly, negative emotion differentiation scores moderated the cross-sectional relationship between stressful life events and depressive symptoms at T1 and it also moderated the impact of T1 stress exposure on prospective T2 depression symptoms 1.5 years later, even after controlling for baseline depression symptoms. In fact, participants with high emotion differentiation scores demonstrated no significant relationship between stress exposure and later depression symptoms. Thus, this study provided evidence at two levels of analysis that emotion differentiation buffers adolescents from the deleterious impacts of stress.

These results were largely replicated in a parallel study by Nook et al. (2021a). This study utilized a year-long intensive longitudinal design that included a smaller number of participants (*N* = 30) but greatly increased within-subjects monitoring. Like Starr et al. (2020a), this study structured measures at two levels. First, participants completed 12 weeks of EMA sampling split into four waves across the year, and although this study used a laboratory-based (rather than EMA-based) measure of emotion differentiation, both positive and negative emotion differentiation scores moderated the concurrent relationship between momentary ratings of perceived stress and depressed affect. Second, participants completed interviews each month in which experimenters coded participants’ exposure to stressful life events and self-report questionnaires assessing depression and anxiety symptoms. Negative emotion differentiation scores also buffered the concurrent association between objective exposure to stressful life events and anxiety symptoms at the monthly level. Similar to the previous study, participants with high emotion differentiation scores actually had no significant relationship between stress and symptoms. Consequently, this study also found that adolescents who are better able to differentiate their emotions appear to be more resilient to stress.

By contrast, Schreuder et al. (2020) did not find a significant moderating impact of emotion differentiation in protecting against psychopathology 1 year later. This lack of replication may have arisen for a few reasons. First, the measure of psychopathology was much broader (i.e., a broad symptom checklist rather than focused assessments of internalizing symptoms; Arrindell and Ettema, 1986). Second, the measure of psychopathology was dichotomized into good vs. bad prognosis, rather than kept continuous, which could have reduced power to find small effects. Third, the measure of stressful life events was administered *retrospectively* at the 1-year follow-up rather than at baseline, potentially clouding measurement of proximal stressors.

Together, these studies offer two points of evidence that emotion differentiation might facilitate adaptive stress coping in adolescents, and one point of evidence against this hypothesis. The replication across the Starr et al. (2020a) and Nook et al. (2021a) papers is compelling given that they differed in methods. The former used a relatively brief EMA measure to assess emotion differentiation and found that it buffered longitudinal changes in depression 1.5 years later in a large sample. The second used a laboratory measure to show the same conceptual finding using an intensive assessment of within-persons fluctuations in internalizing symptoms in a smaller sample. It is also interesting that both found that positive emotion differentiation scores moderated stress-pathology relationships only when measured at the EMA level and not at a monthly or interview-based level, suggesting that the null person-level positive emotion differentiation results reviewed in the previous section might be masking a relationship that exists at a finer level of analysis. It is not obvious why exactly the Schreuder et al. (2020) study did not show a similar replication, but perhaps methodological details (focus on broad prognosis rather than granular changes in internalizing symptoms or timing of the stress measure) may play a role.

Regardless, just as described above, these studies offer glimmers for the adaptive role of emotion differentiation in youth, but a lack of consistent findings suggests that a clear picture is still emerging. Future research should take these discrepant findings into account when scrutinizing the question of whether (and *how*) differentiation protects against psychopathology in youth. Specifically, it seems that continuous (rather than categorical) measures of psychopathology and using concurrent stress measures may be important for detecting this effect. Interestingly, a recent paper using a 4-year longitudinal EMA design in young adults (i.e., college students) found that negative emotion differentiation scores did not moderate the relationship between stress and the emotion regulation strategies participants reported using (Brown et al., 2021). Although it’s possible that the study did not include a measure of the specific strategies that are “active ingredients” in explaining how differentiation buffers the impact of stress (or that these patterns will differ in younger populations), this finding complicates the theoretical picture for how differentiation offers resilience to stress. As described in the Section “Key Questions for the Study of Emotion Differentiation in Youth,” studies like these that work toward clear mechanistic models are sorely needed. Nonetheless, this review highlights what evidence we have assembled so far, as well as the many open questions that must be addressed to arrive at a complete picture of youth emotion differentiation.

## Key Questions for the Study of Emotion Differentiation in Youth

Recent reviews and empirical studies have generated important discussions of key concerns and limitations in the study of emotion differentiation in adults (Trull et al., 2015; O’Toole et al., 2020; Seah and Coifman, 2021; Thompson et al., 2021). In the previous sections, I reviewed papers on youth emotion differentiation and noted that overall, more data are needed for firm conclusions both about how emotion differentiation develops and how it relates to youth mental health. In this section, I expand this discussion to outline what I believe are three of the most pressing issues that loom in our understanding of emotion differentiation beyond the gaps in the literature identified above. Where relevant, I outline how these limitations manifest specifically in the context of youth populations, but many of the concerns raised here also apply to adults. Addressing these and other open questions will be crucial for generating a clear account of emotion differentiation that can spur actionable interventions for improving mental health across development.

### Gaining Consensus on What We’re Measuring and How to Measure It

Eörs Szathmáry once said that linguists “would rather use each other’s toothbrushes than their terminology,” and I fear the same is true of psychologists. Several researchers have already commented on the fact that there are a large set of metrics that fall under the umbrella of “affective dynamics” (e.g., Trull et al., 2015; Dejonckheere et al., 2019; Jeronimus, 2019; Reitsema et al., 2021) and that there may be more effective ways to integrate them into a common framework (Hoemann et al., 2020a). A proliferation of terms is not necessarily a bad thing. If each name matches a distinct construct that is clearly specified at the conceptual and operational level, then our field will have a detailed and precise sense of how affective dynamics function. Unfortunately, though, this is likely not the case, as many of these constructs share conceptual (and even statistical) overlap, and some have gone so far as to say that dynamic measures beyond mean affect contribute little to our understanding of mental health (Grühn et al., 2013; Dejonckheere et al., 2019). So how exactly do we define emotion differentiation in the midst of these manifold constructs, and are our current techniques the best measures for this ability?

Unfortunately, creating a clear taxonomy of constructs is easier said than done. One reason it is difficult to declare which constructs provide unique vs. redundant information is that *context* influences (i) how specific operationalizations map onto the underlying affect dynamics they aim to measure, (ii) how different measures relate to each other, and (iii) how these measures relate to outcomes of interest (Aldao, 2013; Lapate and Heller, 2020; see also Grieve, 2021). This makes it difficult to conclude from any single study—which only captures a single or a small set of contexts—what the taxonomy ought to be. To address this problem, it may be helpful to dissect contextual factors into three levels, specifically study-level, person-level, and situation-level factors. Identifying how factors at each level influence emotion differentiation is an important step in working toward a precise understanding of what any individual’s emotion differentiation score might be capturing and how it should behave.

First, “study-level factors” (i.e., the specific methods through which emotion differentiation is measured in a study) influence emotion differentiation estimates. For example, the predictive power of emotion differentiation measures is influenced by which emotion terms are administered (Erbas et al., 2019), and merely having a longer duration of self-monitoring influences participants’ mean emotion differentiation scores (Widdershoven et al., 2019). Second, “person-level factors” also shape how the same emotion differentiation scores should be interpreted. For example, in Nook et al. (2018) *age* is one such feature, as even if a 5-year-old and a 25-year-old achieve the same score on a laboratory measure of emotion differentiation, these two individuals likely have a radically different profile of emotional experiences. Other person-level variables like vocabulary size or trauma history also likely influence emotion differentiation scores (Nook et al., 2017; Weissman et al., 2020). Third, “situation-level factors” shape how we should interpret emotion differentiation measures. For example, momentary levels of stress influence emotion differentiation scores (Erbas et al., 2018, 2021). Similarly, one would imagine that the literal situations in which one measures participants’ emotional responses would affect emotion differentiation scores and how well they predict psychopathology (e.g., people who avoid fear-inducing situations during sampling periods may never have the opportunity to endorse feeling fear, even though measuring differentiation in these settings might be the most powerful assay of symptom levels). Consequently, further descriptive evidence is needed to fill in the many unknowns of how these factors influence mappings between measures, constructs, and outcomes if we are to develop a replicable and accurate taxonomy for affective dynamics measures that are not confounded by these contextual factors (see Grieve, 2021 for a related argument in linguistics). Once these patterns have been documented, we can work toward a data-driven taxonomy that accurately situates emotion differentiation within the broader network of other constructs.

In this spirit, it seems that there is open space for developing novel measures of emotion differentiation that do not rely on the ICC measure of repeated emotion ratings. Two studies have recently aimed to do just that by coding the granularity of the emotion words participants used when they narrate their emotional reactions (Ottenstein and Lischetzke, 2020; Williams and Uliaszek, 2021). This method aims to directly assess how nuanced or specific participants are when labeling their emotional experiences by coding their language as more undifferentiated (e.g., “bad,” “unpleasant”) or more differentiated (e.g., “frustrated,” “disappointed”) using a coding scheme similar to the Levels of Emotional Awareness Scale (LEAS; Lane et al., 1990). Although this linguistic method offers a face-valid approach to the construct of emotion differentiation, the data surprisingly show that it is not related to the ICC measure and is overall less strongly connected to outcomes (Ottenstein and Lischetzke, 2020; Williams and Uliaszek, 2021). As such, the construct validity of this approach has not been established, and we have little clarity on how actual verbal reports can be used to measure the psychological processes that the ICC method appears to capture. Nonetheless, this line of research affords an opportunity to circumvent some of the context problems raised above, as this method could be applied to standardized vignettes presented in controlled lab environments.

One other looming measurement issue concerns how to measure emotion differentiation in very young populations (and some adult populations) who lack emotion words (see also Shablack and Lindquist, 2019). Emotion vocabulary is constrained to simple words in very young children, and most emotion words are learned across the first ∼10 years of life (Baron-Cohen et al., 2010; Nook et al., 2020; Grosse et al., 2021). Researchers have developed creative designs to test emotion perception in very young children, including pre-verbal infants (e.g., looking time, children’s behavioral responses to maternal facial expressions, and sorting paradigms; Sorce et al., 1985; Widen and Russell, 2008; Wu et al., 2017; Ogren and Johnson, 2020b). Although these paradigms can indeed test whether individuals discriminate between stimuli, they primarily focus on differentiating *emotional stimuli* (e.g., others’ facial expressions, affective utterances), which is not the same as differentiating *one’s own emotions*. Formulating how to measure differentiation of actual emotion experiences in non-verbal individuals is a puzzle in need of a solution.

### Ruling Out Third Variables

A second major threat to a clear understanding of emotion differentiation is the looming specter of untested third variables. These undermine both the question of how emotion differentiation relates to age and how it relates to mental health outcomes. One key third variable has already been identified and discussed: Mean negative affect. In a large study of 1,777 individuals, Dejonckheere et al. (2019) showed that many different affective dynamic measures are either no longer associated with mental health measures (or their associations are drastically reduced in size) when mean negative affect is added as a control variable. Interestingly, emotion differentiation scores remained a significant predictor of psychological well-being even after controlling for mean negative affect. However, the size of the relationship fell from *R*^2^ = 0.04 to *R*^2^ = 0.005, and controlling for mean negative affect rendered the relationship between differentiation scores and depressive symptoms non-significant. This suggests that a large portion of covariance between emotion differentiation scores and well-being is explained merely because people who endorse more negative affect both have worse mental health and have more homogeneous reports of negative emotion.

There are two very different ways to interpret this finding at the conceptual level: (i) emotion differentiation may merely be an artifact of a “true” relationship between heightened negative affect and psychopathology, or (ii) low emotion differentiation may produce elevated endorsements of emotions (e.g., people may anchor ratings on their strongest emotion and then provide similarly strong endorsements for other emotions of the same valence). If the latter is true, controlling for mean negative affect removes true signal produced by emotion differentiation. Adjudicating between these explanations is an important future direction, as one implies that emotion differentiation is epiphenomenal while the other implies that controlling for negative affect metaphorically “throws the baby out with the bathwater.” One way to sidestep this concern is to develop methodological innovations that allow emotion differentiation to be measured separate from daily mean negative affect (e.g., from verbal reports or in response to standardized lab stimuli rather than daily experiences; e.g., Erbas et al., 2014; Nook et al., 2018; Ottenstein and Lischetzke, 2020; Williams and Uliaszek, 2021). Another possibility is to develop experimental methods for shifting emotion differentiation (see “Discussion and Future Directions”) and testing whether this has a causal impact on shifting mean endorsement of negative affect (i.e., empirically testing the mediation model implied above).

Beyond mean negative affect, there are several other third variables that remain underexplored. Overall cognitive abilities (e.g., IQ; Wechsler, 1981; Deary, 2012; Nisbett et al., 2012) are an especially important untestedse of 3rd variables. Both fluid reasoning and verbal knowledge (the two major components of IQ) are associated with emotion and mental health (De Stasio et al., 2014; Opitz et al., 2014; von Salisch et al., 2015; Nook et al., 2017; Miller et al., 2018; Zelazo, 2020), and it stands to reason that they are likely associated with the ability to use words to specifically parse one’s affect. These abilities typically increase across age, but their relations with normative shifts in differentiation scores haven’t been explored in youth samples. As such, it’s possible that cognitive abilities could explain both age-differentiation and mental health-differentiation relationships.

Personality variables (e.g., neuroticism, extraversion, conscientiousness) could similarly serve as third variables for these relationships. For example, highly conscientious individuals may carefully ponder their reactions to each emotion item and provide more nuanced descriptions of their emotions (generating higher emotion differentiation scores) and also be more likely to engage in behavioral habits that promote mental health (e.g., building social relationships; Cohen and Wills, 1985; Bendezú et al., 2019). Two studies reported by Erbas et al. (2014) demonstrated weak relationships between emotion differentiation scores and most personality variables (|*r*| s < 0.06) except for neuroticism (–0.27 < *r*s < –0.17). Although these correlations did not reach significance in either study, they are sizeable enough that future research should seek to ensure they do not confound the research question at hand.

Finally, it is possible that there is a subtle circularity in measures of emotion differentiation and mental health outcomes. Although not truly a “third variable,” it’s important to rule out the possibility that mental health issues could *produce* emotion ratings that would be scored as low differentiation but are actually reflecting the mental health issue itself. For example, fatigue and low motivation are hallmark symptoms of depressive disorders (American Psychiatric Association, 2013). These symptoms could produce low engagement in the research study, especially if they require many repeated emotion ratings. A lack of engagement could produce similar numerical ratings across scales, a high ICC across emotions, and ultimately low differentiation scores. Consequently, if participants with low motivation in fact experienced highly differentiated emotional experiences but provided homogenous emotion ratings due to a lack of task engagement, this could produce a spurious correlation between emotion differentiation scores and depression symptoms.

We also know that people with mental health difficulties are more likely than controls to (i) make decisions that increase their exposure to stressful situations and (ii) be exposed to systemic adversities (e.g., racism, ostracism, low socioeconomic resources; Adrian and Hammen, 1993; Meyer, 1995; Cole et al., 2006; Sheridan and McLaughlin, 2014; Wadsworth et al., 2016; Vaid and Lansing, 2020). Both of these pressures may shift the typical situations that these individuals are in, potentially generating different profiles of emotion responding in which emotions are naturally co-experienced more intensely than for individuals without these experiences. If so, this could similarly create a natural bias in emotion differentiation score computations that also generate a circular result. The possibility that measures of emotion differentiation may tap externalities of mental health difficulties—rather than the individual’s ability to differentiate emotions—is a thorny issue that similarly requires conceptual and methodological innovation to address.

One important note to keep in mind when pursuing these third variables is that it is important to differentiate whether they are operating as true deflationary confounds versus potential mechanisms in a more complex model. For example, it is possible that verbal knowledge operates *either* as a true deflationary confound or as an interesting variable in a mechanistic explanation of the relationship between emotion differentiation and mental health. Having a larger vocabulary may produce more differentiated emotional experiences and facilitate mental health *without* there being any relationship between differentiation and mental health. Conversely, emotion differentiation might actually *explain why* vocabulary is associated with mental health (i.e., people with larger vocabularies can use more specific terms to label their emotions, boosting regulation and ultimately mental health). This distinction becomes especially interesting in a developmental context, where language may contribute to growths in emotion conceptualization (Nook et al., 2017; Hoemann et al., 2019, 2020b; Nook and Somerville, 2019). Similarly, as hinted above, mean negative affect may confound the relationship between differentiation and health, or it may *mediate* this relationship. Studies that engage in careful and thorough adjudication between these different possibilities are needed.

### Clarifying Mechanisms and Developing Causal Models

At present, there are not clearly specified causal models that explain either (i) how emotion differentiation develops or (ii) why emotion differentiation is associated with mental health. On the one hand, this is understandable given the developmental stage of our science. The first formulation of emotion differentiation was just 20 years ago (Barrett et al., 2001) and the current review revealed only seven papers focusing on emotion differentiation in youth. It is consequently understandable for us to still be in a *descriptive* stage of scientific discovery, in which scientists focus merely on describing the general properties of emotion differentiation (e.g., its correlates). This stage has generated enthusiasm in the field, and above I advocated for continued effort at the descriptive stage of discovery, given the number of open questions regarding how to conceptualize and measure emotion differentiation. However, the world is desperate for improved techniques to bolster mental health (Kazdin and Blase, 2011; Sawyer et al., 2012; Kazdin and Rabbitt, 2013) and although intervening on emotion differentiation could contribute to mental health efforts, fully realizing this goal will require that we move beyond mere description into formulation of precise theories and thoroughly tested causal models (Eronen and Bringmann, 2021; Robinaugh et al., 2021).

The findings from Nook et al. (2018) summarized above provide an initial step in formulating a model of how emotion differentiation develops, but it is by no means complete. First, it only captures variation across ages 5–25, leaving the early ontogeny of this process unclear. Are emotions at even younger ages also clearly understood in a mutually exclusive fashion? Emotion representation processes from birth to age 5 need to be clarified for a complete theory of emotion differentiation development (see Ogren and Johnson, 2020a; Ruba and Repacholi, 2019 for recent reviews on early emotion development). Second, we have very little insight into the psychological and neural *mechanisms* that explain why these two processes would unfold as they do. Do children indeed *experience* only one emotion at a time, or is this effect produced by how they respond to rating scales? If it does indeed reflect their actual emotional experiences, is this because their actual physiology only triggers affect for one emotion at a time or is it because they have a higher-level belief that leads them to only parse their affect into one emotion type at a time?

Answering these questions will also likely elucidate the mechanisms underlying why adolescence is a period of low emotion differentiation: Does this emerge because of changes in the physiological generation of affect (i.e., hormonal or neural changes producing “messier” affective signals), changes in higher-order beliefs (e.g., the recognition that emotions can occur leading to more complex constructions of what emotions they are feeling), or perhaps some other psychological change (e.g., growing mentalizing skills that could influence how stimuli are interpreted or protracted developments in how the emotion words used in these tasks are interpreted)? Working toward a multilevel causal model may also clarify whether the normative decrease in differentiation across age reflects maladaptive shifts that expose adolescents to increase risk for psychopathology or whether they reflect natural adaptations that overall promote well-being during this developmental period.

The field has also assembled a similarly reasonable, though underspecified, model for why emotion differentiation scores are associated with improved mental health revolving around the idea that being able to specifically identify one’s emotions facilitates (i) more effective regulation of negative emotions and/or (ii) selection of more adaptive regulatory strategies. However, it is largely unclear *why* differentiation would afford more effective or healthier regulation: What psychological beliefs, processes, abilities, or computations explain these relations? Research on language and emotion suggests that activating emotion words can influence how people construct emotional experiences (Lindquist et al., 2006; Gendron et al., 2012; Lindquist et al., 2015; Nook et al., 2015; Satpute et al., 2016), that merely labeling emotions can reduce the intensity of those emotions (Lieberman et al., 2011; Kircanski et al., 2012; Torre and Lieberman, 2018), and that people who lack abilities to verbalize their emotions also struggle to effectively manage them (Taylor and Bagby, 2004; Leweke et al., 2011; Weissman et al., 2020). These lines of data suggest that applying specific emotion language should facilitate later regulation. However, tight empirical investigations have actually found the opposite: Labeling emotions makes subsequent regulation *less* effective (Nook et al., 2021b), and labeling emotions using many emotion words leads people to select more *maladaptive* regulatory strategies than if they had used just a few emotion words (Vine et al., 2019). There are certainly ways to iron out the logic to make these findings fit (e.g., perhaps precise labeling boosts regulation at longer time horizons than these experiments tested?), but right now they run counter to theoretical intuitions that emotion language boosts regulation. As such, close scrutiny is needed to more fully understand how the simple act of labeling our emotions affects regulation if we hope to address the bigger question of why differentiation is associated with mental health.

There are several other aspects of this theoretical model that can be further refined, and addressing these open questions is likely to require collaboration across psychological subdisciplines. For instance, even though a handful of studies have used neuroimaging or psychophysiological approaches to study emotion differentiation (Lee et al., 2017; Wang et al., 2020a,b; Hoemann et al., 2021), the biological mechanisms that explain why emotion differentiation boosts regulation remain largely unclear. Additionally, the clinical question of *multifinality* has yet to be addressed (e.g., why are some people with low emotion differentiation prone to alcohol use, while others engage in non-suicidal self-injury?). Although it seems that low emotion differentiation generates *transdiagnostic* risk, how exactly does it do so, and what forces push someone with low differentiation to experience specific forms of pathology? Emerging models of psychopathology are shifting away from a categorical model in which specific illnesses have unique discrete essences toward frameworks where syndromes reflect underlying dimensions of dysregulation and/or networks of interacting symptoms (McNally et al., 2015; Kotov et al., 2017). Incorporating these theoretical approaches into studies on emotion differentiation and including samples that are selected to include elevated levels of psychopathology could help address these questions. Finally, potential *interpersonal* mechanisms that might explain the benefits of emotion differentiation have only begun to be explored. For example, emotion differentiation may facilitate empathy (Erbas et al., 2016; Israelashvili et al., 2019) and accurate prediction of others’ emotions is associated with relationship quality (Zhao et al., 2020), potentially explaining why low emotional awareness is associated with worse mental health in adolescent girls (Weissman et al., 2020).

Answering these open questions through a specific, testable, and multilevel theory (that also rules out the third-variables noted above) will greatly advance the translational impact of research on emotion differentiation. To summarize this section, this would ideally lead to a clear model with two parts. First, a precise explanation of *what psychological processes* produce any given individual’s emotion differentiation score taking into account person-level factors (e.g., age), study-level factors (e.g., the emotion words they rated), and situation-level factors (e.g., the settings in which they reported on their emotions). Second, a precise explanation of how these psychological processes then produce their level of psychological functioning. At present, both parts of this model are not clearly articulated, rendering low emotion differentiation scores something of akin to a “maintenance required” light in a car: Low scores suggest that *something* might not be quite right with a person’s level of psychological functioning, but we don’t really know what mechanisms are producing the scores or why they have deleterious impacts. Moving from this “maintenance required” indicator stage to a clear understanding of the mechanistic psychological components that are operating “under the hood” is an exciting and important horizon of future research.

## Discussion and Future Directions

Scientific understanding of youth emotion differentiation is in its infancy, and this review has unearthed many unanswered questions (see Box 1). Below I outline general guidelines that can help future researchers interested in advancing this understanding. In particular, I describe strategies for maximizing reproducibility, addressing the open questions raised above, and working toward interventions.

Beyond the obvious advice of using sample sizes that are well-powered to reliably detect small-to-medium effects (Richard et al., 2003; Open Science Collaboration, 2015), open science practices offer clear strategies for enhancing replicability of findings (Kathawalla et al., 2021). Preregistration—where researchers commit to their data collection procedures, inclusion/exclusion criteria, analytic plan, and hypotheses—reduce the possibility that published findings include only cherry-picked significant associations that may actually be false-positives. Individual replication studies or planning internal replications within papers (either by conducting additional studies or through a split-sample approach) can generate additional data for testing the stability of effects. Data sharing can allow future researchers to verify results as well as compile datasets to assess replicability and test moderators that might explain heterogeneous effects across studies. Indeed, cross-group collaborations in which datasets are shared across research labs, as is done in genetics (e.g., Smeland et al., 2019), could greatly speed progress on the many open questions raised in this review. Fortunately, researchers have begun to compile and publicly share EMA datasets^1^. All of these open science practices are likely to pay dividends in the future. Additional considerations for maximizing reproducibility in developmental studies include using a wide age range, measuring potential explanations for age-related effects, and ensuring that study procedures are suitable for diverse ages (e.g., controlling relevance of stimuli).

The open questions raised above can help organize and inform future research on emotion differentiation in development. However, it should be assumed that *substantial* additional information is needed to address these questions, and a single study is unlikely to accomplish this task. As such, scientists should consider issues of measurement at both the conceptual and methodological levels when designing studies. For example, consider what other affective dynamics measures can be extracted and have a plan for analyzing how these measures relate (e.g., through factor or network analyses; Lange et al., 2020). Also consider how selection of specific emotion terms should either intentionally match prior research or include a larger set to test whether the emotions that are measured influence the results. Studies should also intentionally focus on the third variables highlighted previously, testing both whether they confound relationships between emotion differentiation and age as well as relations between emotion differentiation and mental health. However, analyses should explore the multiple pathways through which these third variables could influence results (i.e., as confounds that create spurious relationships or as mechanisms in a larger model).

*Longitudinal* and *experimental* paradigms will likely prove invaluable in disentangling the influence of potential third variables and working toward causal models. For instance, demonstrating that vocabulary development longitudinally precedes changes in emotion differentiation can help test the causal direction of influence. Similarly, using paradigms that facilitate or interfere with access to emotion concepts (see Halberstadt, 2005; Lindquist et al., 2006; Gendron et al., 2012; Nook et al., 2015; Barker et al., 2020; Satpute et al., 2020) could hone in on the causal influence of emotion differentiation on downstream processes. Relatedly, developing effective interventions that boost emotion differentiation can then be used to causally test mechanisms thought to explain both (i) the genesis of strong differentiation skills and (ii) how differentiation improves mental health.

However, developing these interventions would benefit from incorporating emerging trends in intervention science. Ideally, these interventions would borrow from mechanisms outlined in basic research as well as theoretical models. For example, they might target enhancing mindfulness to the affect generated during different emotional experiences (Van der Gucht et al., 2019), equipping individuals with emotion vocabulary (Nook et al., 2017), or educating individuals in how to have refined conceptual representations of different emotions (Hoemann et al., 2019). Conversely, studies can reverse-engineer potential intervention targets and mechanisms of influence by analyzing if and how existing interventions (e.g., psychotherapy) increases differentiation (Linehan, 1993; Barlow et al., 2011). Intervention scientists have developed clear guidelines for structuring these experiments to maximize knowledge not only concerning *what* interventions work but *why* they work (Kazdin, 2007; Ng and Weisz, 2016). In fact, a recent paper shows that Emotion Regulation Therapy (Mennin, 2004; Mennin and Fresco, 2015) improves negative emotion differentiation, setting the stage for a line of research that dissects the mechanisms underlying this effect (Mikkelsen et al., 2021). Finally, the notion of single-session interventions have recently gained heightened interest (Schleider and Weisz, 2017), motivating the question of what *dose* of intervention is needed effectively shift differentiation and mental health.

Though challenging, it seems apparent that partnerships between affective, developmental, and clinical psychologists could produce a promising set of tools for explaining, detecting, treating, and potentially even preventing psychopathology. Indeed, given the number of open questions, it seems wise to maintain a sense of patience, optimism, and collaboration in this pursuit for clarity. The current review aims to summarize both what we do and do not know about emotion differentiation in youth, with the ultimate goal of achieving a clear science of how emotions go awry and what we can do to prevent these experiences.

## Conclusion

Although emotion differentiation is consistently associated with mental health in adults, there are substantial open questions concerning *how* this ability arises and *why* it is associated with well-being. Taking a developmental perspective on both questions offers a powerful opportunity for disentangling potential causal processes and developing strategies for intervening early to minimize the public health burden of psychopathology. Only by addressing open questions concerning measurement and looming third variables can we develop a clear and useful theoretical model.

## Author Contributions

EN developed the manuscript’s thesis, conducted the literature review, and wrote the manuscript.

## Conflict of Interest

The author declares that the research was conducted in the absence of any commercial or financial relationships that could be construed as a potential conflict of interest.

## Publisher’s Note

All claims expressed in this article are solely those of the authors and do not necessarily represent those of their affiliated organizations, or those of the publisher, the editors and the reviewers. Any product that may be evaluated in this article, or claim that may be made by its manufacturer, is not guaranteed or endorsed by the publisher.

## Footnotes

1
https://emotedatabase.com/
